# TLR5在不同非小细胞肺癌细胞株的表达及其活化机制的初步探讨

**DOI:** 10.3779/j.issn.1009-3419.2015.01.02

**Published:** 2015-01-20

**Authors:** 辉 周, 媚 罗, 亦戈 文, 安迪 马, 永忠 罗, 青 易, 建华 陈, 玲 肖

**Affiliations:** 1 410013 长沙，湖南省肿瘤医院，中南大学湘雅医学院附属肿瘤医院 Hunan Cancer Hospital, the Affiliated Cancer Hospital of Xiangya School of Medicine, Central South University, Changsha 410013, China; 2 410078 长沙，中南大学国家遗传学重点实验室 State Key Laboratory of Medical Genetics Central South University, Changsha 410078, China; 3 410013 长沙，中南大学基础医学院组织学与胚胎学系 Department of Histology and Embryology, School of Basic Medical Sciences, Central South University, Changsha 410013, China

**Keywords:** 肺肿瘤, TLR5, 鞭毛蛋白, 信号通路, Lung neoplams, TLR5, Flagellin, Signaling pathway

## Abstract

**背景与目的:**

已有的研究表明：Toll样受体5（toll-like receptor 5, TLR5）在肿瘤起始和发展中发挥重要作用。我们前期研究发现，TLR5在非小细胞肺癌（non-small cell lung cancer, NSCLC）组织中高表达，但其在NSCLC高表达后的信号通路活化情况的研究并不多见。本研究旨在探讨TLR5在不同NSCLC细胞株上的表达，及其在NSCLC细胞中活化的机制。

**方法:**

用免疫荧光、RT-PCR和Western blot方法检测TLR5在三种不同NSCLC细胞株中的表达。分别用0 μg/mL、0.01 μg/mL、0.1 μg/mL、1 μg/mL、5 μg/mL、10 μg/mL的鞭毛蛋白刺激，用NF-κB荧光素酶报告基因质粒瞬时转染后，检测细胞内NF-κB荧光素酶的活性。选择TLR5表达最高的SPC-A-1细胞株为实验对象，选择0.1 μg/mL的鞭毛蛋白，分别用0 μg/mL、0.01 μg/mL、0.1 μg/mL、1 μg/mL、10 μg/mL的TLR5抗体抑制通路活化，检测细胞内NF-κB荧光素酶的活性，验证TLR5活化通路。构建TLR5-shRNA，转染SPC-A-1细胞48 h后，以0.1 μg/mL浓度鞭毛蛋白分别刺激SPC-A-1细胞及转染的SPC-A-1细胞，在刺激0 min、10 min、30 min、60 min，用Western blot方法比较TLR5信号通路因子p-IKBα、p-ERK1/2、p-JNK、IKBα、ERK1/2的变化。

**结果:**

TLR5在肺腺癌细胞株SPC-A-1中呈高表达，且主要表达在细胞膜上。三种细胞株中SPC-A-1细胞NF-κB荧光素酶的活性最高，呈浓度依赖性，0.1 μg/mL鞭毛蛋白即可明显增强NF-κB荧光素酶的活性（*P* < 0.05）；而SPC-A-1细胞内NF-κB荧光素酶的活性可被TLR5抗体抑制，与TLR5抗体浓度负相关（*P* < 0.05）。与0 min相比较，SPC-A-1细胞内p-IKBα、p-ERK1/2、p-JNK水平在鞭毛蛋白刺激10 min即明显增高，30 min达到高峰，60 min开始下降（*P* < 0.05），且与10 min和60 min组相比，p-IKBα、p-ERK1/2、p-JNK水平在30 min增高（*P* < 0.05）；而IKBα、ERK1/2的水平无明显变化（*P* > 0.05）。以适合浓度鞭毛蛋白刺激转染的SPC-A-1细胞，p-IKBα、p-JNK蛋白均未检出，IKBα、ERK1/2蛋白的水平无明显变化（*P* > 0.05），p-ERK1/2蛋白水平随着时间延长明显增高（*P* < 0.05）。

**结论:**

外源性配体鞭毛蛋白可激活NSCLC细胞株TLR5蛋白，启动下游信号通路，可能与NSCLC的发生发展有关。

## Abstract (en)

**Background and objective:**

It has been proven that toll-like receptor 5 (TLR5) plaied an important role in the development of tumor. In our previous study, we found that the expression of TLR5 was remarkably higher in non-small cell lung cancer (NSCLC) tissues than that in normal tissues, but the activation of TLR5 signaling pathway in NSCLC was still unknown. The aim of this study is to investigate the expression of TLR5 in different types of NSCLC cell lines, and analyze the activity of the signaling pathway after stimulated by its specific exogenous ligand flagellin.

**Methods:**

The TLR5 protein was detected by immunofluorescence and Western blot in three kinds of NSCLC cell lines, and the TLR5 mRNA was detected by RT-PCR. Select the cell line of TLR5 highest expression as the research object, and select the suitable concentration of flagellin. NF-κB luciferase activity was detected to validate the TLR5 activation pathway through inhibitory signaling pathways by 0 μg/mL, 0.01 μg/mL, 0.1 μg/mL, 1 μg/mL, 10 μg/mL TLR5 antibody. The chosen cell line was transfected by TLR5 shRNA plasmid, and p-IKBα, IKBα, p-ERK1/2, ERK1/2 and p-JNK of untrasfected and transfected cells were detected in the activity of TLR5 signaling pathway by Western blot at 0 min, 10 min, 30 min and 60 min, respectively.

**Results:**

The expression of TLR5 was the highest in the lung adenocarcinoma cell line SPC-A-1 by immunofluorescence, mainly expressed on the cell membrane. NF-κB luciferase activity of SPC-A-1 cells was the highest, and the activity was increased in a dose-dependent manner. 0.1 μg/mL flagellin could significantly increase the NF-κB luciferase activity (*P* < 0.05), while its activity could be inhibited by the TLR5 antibody in a negative correlation. Treated by 0.1 μg/mL flagellin, compared with that of 0 min group, the levels of p-IKBα, p-ERK1/2, p-JNK of SPC-A-1 cells increased significantly after 10 min, reached the peak at 30 min, and declined at 60 min (*P* < 0.05). Compared with that of 10 min and 60 min group, the levels of p-IKBα, p-ERK1/2, p-JNK significantly increased at 30 min (*P* < 0.05). While the levels of IKBα, ERK1/2 at 0 min, 10 min, 30 min and 60 min had no significant changes (*P* > 0.05). SPC-A-1 cells transfected TLR5-shRNA were also stimulated by flagellin (0.1 μg/mL). At 0 min, 10 min, 30 min and 60 min, p-IKBα and p-JNK proteins could not be detected, and the levels of IKBα and ERK1/2 had no significant changes (*P* > 0.05), but the levels of p-ERK1/2 significantly increased as time went on (*P* < 0.05).

**Conclusion:**

Exogenous ligand flagellin can activate TLR5 protein in NSCLC cell lines and initiate downstream signaling pathways. It may be relative to the development of NSCLC.

肺癌是最常见的恶性肿瘤，发病率高，生存率低，预后差，居恶性肿瘤死亡率首位^[1]^。非小细胞肺癌（non-small cell lung cancer, NSCLC）约占肺癌的80%-85%，研究^[2]^发现慢性炎症与肿瘤发生相关。Toll样受体5（toll-like receptor 5, TLR5）是toll样受体家族中的一员，是一类最具有特征性的模式受体分子，能募集多种配体蛋白，激发信号转导，导致一些特异性转录因子的活化。细菌的鞭毛蛋白（Flagellin）作为TLR5特异性的外源性配体，TLR5与其结合后具有保护性的抗炎作用，其可能在肿瘤异常表达。我们前期研究^[3]^发现，与正常肺组织比较，TLR5在NSCLC组织中高表达，且与吸烟分化程度呈正相关，但有关TLR5在NSCLC高表达后的信号通路活化情况的研究并不多见。本研究旨在探讨不同NSCLC细胞株中TLR5表达情况，寻找较好的实验模型，并探讨其信号通路活化的机制。

## 1 材料与方法

### 1.1 材料

#### 1.1.1 细胞株

人肺腺癌细胞株SPC-A-1购于中科院上海细胞研究所；人肺腺癌细胞株A549（cat# CCL-185），人肺鳞癌细胞株SK-MES-1（cat# HTB-58）均购自美国菌种保藏中心（American Type Culture Collection, ATCC）。

#### 1.1.2 主要试剂和耗材

DMEM培养基、FBS、胰酶均购自美国ATCC公司；Triton X-100购自美国Genview公司；反转录试剂盒、PCR Mix试剂盒均购自加拿大Fermentas（MBI Fermentas）分子生物技术公司；Tris-Base、10%SDS均购自美国Sigma公司；TEMED购自上海生工生物工程股份有限公司；天然膜蛋白抽提试剂盒ProteoExtract^TM^（M-PEK）购自德国MERCK公司（中国）；Total protein Extraction试剂盒购自美国ProMab公司；NucBuster^TM^ Protein Exaction Kit购自德国MERCK公司（中国）；Mouse GAPDH antibody、Goat Anti Rabbit IgG/HRP购自美国Santa Cruz生物公司；Rabbit Anti Goat IgG/HRP购自中杉金桥技术有限公司（北京）；Goat Anti Mouse IgG/HRP、Goat Anti Mouse IgG+A+M (H+L)/HRP均购自美国Zymed Laboratories公司；Goat anti-rat IgG-HRP、Rabbit Anti-Sheep IgG/HRP均购自美国Santa Cruz生物技术公司；NF-κB荧光素酶报告基因质粒购自江苏碧云天技术研究所。shRNA空载体pLKO.3G购自Addgene公司；Lipofectamine^TM^ 2000试剂、Opti-MEM^®^低血清培养基均购自美国Invitrogen公司。

#### 1.1.3 引物

见表 1。

**1:** 引物 Primer

Primer	Sequence(5'- > 3')		Products (bp)	Annealing temperature (℃)
TLR5	Upstream	CCTTAGAGATGGCTGGTGCC	89	60
	Downstream	CCACCACCATGATGAGAGCA		
TLR5: toll-like receptor 5.				

### 1.2 方法

#### 1.2.1 TLR5在不同NSCLC细胞系的表达

免疫荧光观察不同NSCLC细胞株TLR5蛋白表达，Trizol法提取细胞的RNA，RT-PCR检测不同肺癌细胞株TLR5 mRNA的水平，并用琼脂糖凝胶电泳检测。通过gel-pro 6.0灰度扫描软件分析各细胞株TLR5的表达状况。Western blot检测TLR5蛋白表达水平。

#### 1.2.2 TLR5与NF-κB信号通路的关系

分别用0 μg/mL、0.01 μg/mL、0.1 μg/mL、1 μg/mL、5 μg/mL、10 μg/mL的鞭毛蛋白刺激，用NF-κB荧光素酶报告基因质粒瞬时转染三种细胞株，检测细胞内NF-κB荧光素酶的活性。选择出TLR5表达最高的细胞株作为研究对象，选择适合浓度的鞭毛蛋白，分别用0 μg/mL、0.01 μg/mL、0.1 μg/mL、1 μg/mL、10 μg/mL的TLR5抗体抑制通路活化，检测细胞内NF-κB荧光素酶的活性，验证TLR5活化通路。

#### 1.2.3 TLR5信号通路磷酸化水平检测

用适合浓度的鞭毛蛋白刺激SPC-A-1细胞，分别在0 min、10 min、30 min和60 min用Western blot方法检测TLR5活化后作用通路中的p-IKBα、IKBα、p-ERK1/2、ERK1/2、p-JNK等分子的变化。构建靶向*TLR5*基因的shRNA质粒，用脂质体法体外转染SPC-A-1细胞，48 h后用相同浓度鞭毛蛋白刺激，Western blot法在相同的四个时间点检测对TLR5信号通路分子的变化（β-actin作为内参）。

### 1.3 统计学方法

数据分析采用SPSS 17.0软件（美国）进行，计量数据表示为均数±标准差（Mean±SD）。计量资料使用*t*检验，计数资料使用*χ*^2^检验，*P* < 0.05认为差异有统计学意义。

## 2 结果

### 2.1 TLR5在不同NSCLC细胞系的表达

免疫荧光显示TLR5蛋白在肺腺癌细胞株SPC-A-1中呈高表达，且主要表现在细胞膜上，在细胞质中也表达（图 1A）。在两种肺腺癌细胞株均可检测到TLR5 mRNA表达（SPC-A-1和A549细胞中TLR5 mRNA相应对表达量分别为0.386±0.022、0.293±0.018），而肺鳞癌细胞株SK-MES-1未检出（*P* < 0.05）（图 1B）。Western blot检测结果显示肺腺癌细胞株SPC-A-1和A549中均有TLR5蛋白表达（SPC-A-1和A549细胞中TLR蛋白相对表达量分别为0.446±0.023、0.195±0.011），而且SPC-A-1细胞株中TLR5蛋白的水平明显高于A549细胞株（*P* < 0.05），肺鳞癌细胞株中未检出TLR5蛋白（图 1C，图 1D）。

**1:**
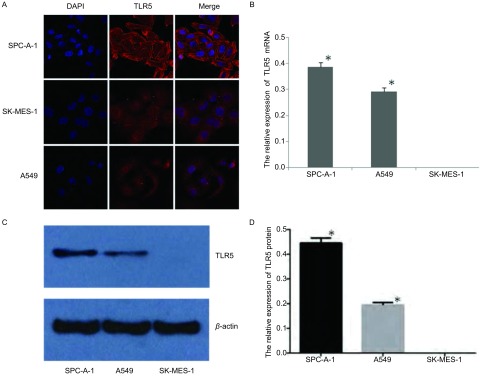
TLR5在不同NSCLC细胞株中的表达。A：免疫荧光显示TLR5在三种NSCLC细胞株中的表达，其主要表达在细胞膜上；B：三种NSCLC细胞株中的表达TLR5 mRNA的相对表达量，与SK-MES-1细胞株相比，^*^P < 0.05；C、D：Western blot检测三种NSCLC细胞株中TLR5蛋白的表达及其相对表达量，与SK-MES-1细胞株相比，^*^P < 0.05。 Expression of TLR5 in three different NSCLC cell lines. A: TLR5 expression in three kinds of NSCLC cell lines by immunofluorescence, which is mainly expressed in the cell membrane; B: The relative expression of TLR5 mRNA in three kinds of NSCLC cell lines; compared with that of SK-MES-1 cell lines, ^*^P < 0.05; C, D: the expression of TLR5 protein in three kinds of NSCLC cell lines and their relative expressions detected by Western blot; compared with that of SK-MES-1 cell lines, ^*^P < 0.05. NSCLC: non-small cell lung cancer.

### 2.2 TLR5与NF-κB信号通路的关系（不同浓度Flagellin刺激）

用NF-κB荧光素酶报告基因质粒瞬时转染后，检测细胞内荧光素酶的活性。随着鞭毛蛋白浓度的增加，SPC-A-1细胞内NF-κB荧光素酶的活性增强，且呈浓度依赖性，0.1 μg/mL鞭毛蛋白即可明显增强NF-κB荧光素酶的活性（*P* < 0.05）；而在A549和SK-MES-1细胞中，NF-κB荧光素酶的活性变化不如SPC-A-1细胞明显（表 2，图 2A）。SPC-A-1细胞内NF-κB荧光素酶的活性可被TLR5抗体抑制，随着TLR5抗体浓度的增加，NF-κB荧光素酶的活性明显降低（*P* < 0.05），与TLR5抗体浓度负相关（表 3，图 2B）。

**2:** Flagellin刺激后，NSCLC细胞内NF-*κ*B荧光素酶相对活性 The relative activity of NF-*κ*B luciferase of NSCLC cells after stimulated by flagellin

	Flagellin (*μ*g/mL)					
	0	0.01	0.1	1	5	10
SPC-A-1	1	2.00±0.09	3.20±0.15	8.00±0.45	18.00±0.92	22.00±1.22
A549	1	1.60±0.08	2.20±0.12	3.60±0.19	6.70±0.34	7.30±0.18
SK-MES-1	1	1.30±0.07	1.10±0.06	1.50±0.08	2.50±0.08	2.40±0.11

**2:**
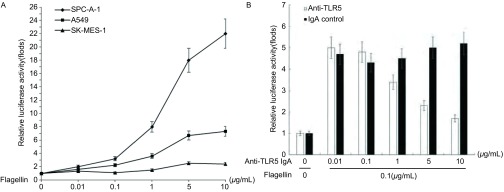
Flagellin刺激后，细胞内NF-*κ*B荧光素酶活性。A：三种NSCLC细胞株的NF-*κ*B荧光素酶活性；B：加入TLT5抗体拮抗后，SPC-A-1细胞内NF-*κ*B荧光素酶活性。 The relative activity of NF- kappa B luciferase of the cells when stimulated by flagellin. A: the relative activity of NF-*κ*B luciferase of three kinds of NSCLC cell lines; B: After added with TLR5 antibody, the relative activity of NF-*κ*B luciferase of SPC-A-1 cells.

**3:** 加入TLR5抗体，Flagellin刺激SPC-A-1细胞，其细胞内NF-*κ*B荧光素酶相对活性 The relative activity of NF-*κ*B luciferase of SPC-A-1 cells stimulated by flagellin when added with TLR5 antibody

	Flagellin (*μ*g/mL)					
	0	0.01	0.1	1	5	10
Anti-TLR5	1	5.00±0.24	4.80±0.21	3.40±0.15	2.30±0.11	1.70±0.09
IgA control	1	4.70±0.22	4.30±0.19	4.50±0.21	5.00±0.26	5.20±0.25
TLR5: toll-like receptor 5.						

### 2.3 TLR5信号通路磷酸化水平检测

选取0.1 μg/mL鞭毛蛋白处理SPC-A-1细胞，分别在作用0 min、10 min、30 min、60 min后，收集细胞进行Western blot检测。结果显示，与0 min相比较，p-IKBα、p-ERK1/2、p-JNK水平在鞭毛蛋白刺激10 min即明显增高，30 min达到高峰，60 min开始下降（*P* < 0.05），且与10 min和60 min相比，p-IKBα、p-ERK1/2、p-JNK水平在30 min明显增高（*P* < 0.05）；而IKBα、ERK1/2的水平在0 min、10 min、30 min、60 min均无明显变化（*P* > 0.05）（表 4，图 3）。

**4:** 鞭毛蛋白刺激后，TLR5信号通路分子相对表达量 The relative expression of TLT5 signaling pathway molecules after stimulated by flagellin

	Time (min)			
	0	10	30	60
p-IKB*α*	0	0.279±0.013	0.363±0.019	0.208±0.011
IKB*α*	0.641±0.031	0.627±0.032	0.608±0.031	0.638±0.032
p-ERK1/2	0.082±0.005	0.449±0.024	0.614±0.033	0.366±0.017
ERK1/2	1.029±0.044	1.057±0.051	1.014±0.048	1.043±0.049
p-JNK	0.198±0.009	0.576±0.029	0.815±0.036	0.411±0.022

**3:**
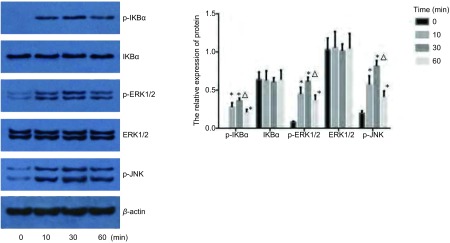
鞭毛蛋白刺激后，TLR5信号通路分子磷酸化水平。*:与0 min组相比，*P* < 0.05；△：与其他组相比，*P* < 0.05。 The phosphorylation of TLT5 signaling pathway molecules after stimulated by flagellin. *: compared with that of 0 min group, *P* < 0.05; △: compared with the other groups, *P* < 0.05.

### 2.4 TLR5-shRNA转染效率及转染后TLR5的表达情况

在转染48 h后，在荧光显微镜下可见空质粒对照组和TLR5-shRNA组被转染SPC-A-1细胞数多，转染效果高。TLR5-shRNA转染SPC-A-1细胞48 h后，与空白对照组（TLR5蛋白相对表达量为0.382±0.017）相比，TLR5-shRNA组细胞TLR5表达（TLR5蛋白相对表达量为0.118±0.008）明显受到抑制（图 4）。

**4:**
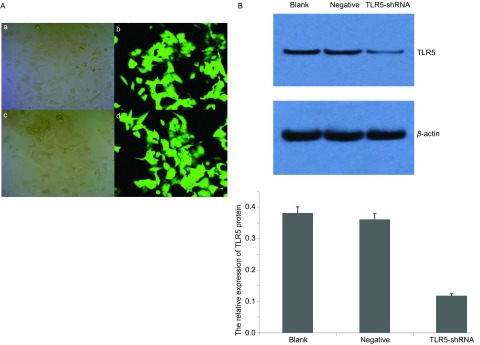
TLR5 shRNA转染SPC-A-1细胞及TLR5表达。A：a:空载体质粒转染组，200×；b:空载体质粒转染组，荧光，400×；c: TLR5-shRNA转染组，200×；d：TLR5-shRNA转染组，荧光，400×；B：Western blot检测。 SPC-A-1 cells transfected with TLR5-shRNA and the expression of *TLR5* genes. A: a: SPC-A-1 cells transfected with vector, 200×; b: SPC-A-1 cells transfected with vector under the fluorescence microscope, 400×; c: SPC-A-1 cells transfected with TLR5-shRNA, 200×; d: SPC-A-1 cells transfected with TLR5-shRNA under the fluorescence microscope, 400×; B: Detected by Western blot.

### 2.5 Flagellin对TLR5-shRNA SPC-A-1细胞TLR5信号通路的影响

TLR5-shRNA转染SPC-A-1细胞48 h后，加入Flagellin（0.1 µg/mL），用Western blot法分别在0 min、10 min、30 min、60 min检测信号通路相关蛋白的表达。结果显示，在四个不同的作用时间点，IKBα、ERK1/2蛋白的水平无明显变化（*P* > 0.05），p-ERK1/2蛋白水平随着时间延长明显增高（*P* < 0.05），而p-IKBα、p-JNK蛋白均未检出（表 5，图 5）。

**5:** Flagellin刺激TLR5-shRNA SPC-A-1细胞不同时间点TLR5信号通路分子相对表达量 The relative expression of TLT5 signaling pathway molecules at different time after TLR5-shRNA SPC-A-1 cells were stimulated by flagellin

	Time (min)			
	0	10	30	60
p-IKB*α*	0	0	0	0
IKB*α*	0.398±0.019	0.412±0.021	0.449±0.023	0.427±0.022
p-ERK1/2	0.116±0.006	0.222±0.012	0.334±0.015	0.447±0.023
ERK1/2	0.990±0.049	0.965±0.045	1.010±0.052	1.005±0.048
p-JNK	0	0	0	0

**5:**
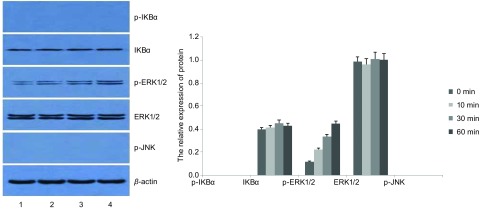
Flagellin刺激TLR5 shRNA SPC-A-1细胞不同时间点TLR5通路分子的表达。1：0 min；2：10 min；3：30 min；4：60 min。 The relative expression of TLT5 signaling pathway molecules at different time after TLR5-shRNA SPC-A-1 cells were stimulated by flagellin. 1: 0 min; 2: 10 min; 3: 30 min; 4: 60 min.

## 3 讨论

TLRs在机体粘膜免疫、肿瘤起始和发展中发挥重要作用，不仅在免疫细胞上表达，也广泛表达于正常上皮细胞和胃癌等多种肿瘤细胞^[4, 5]^。我们前期实验发现TLR5蛋白在NSCLC组织中表达明显增高，在不同组织学类型的表达阳性率为鳞癌65%、腺癌82.1%、鳞腺癌50%、其他类型20%，其中TLR5在腺癌中表达阳性率最高。为了进一步研究其高表达的意义和作用机制，挑选合适的细胞株作为研究对象，我们分别检测TLR5在两种人肺腺癌细胞株SPC-A-1、A549细胞和人肺鳞癌细胞株SK-MES-1中的表达情况。免疫荧光、RT-PCR和Western blot结果表明在人肺鳞癌细胞株SK-MES-1中未检测到TLR5表达，两种不同NSCLC腺癌细胞株中均有TLR5的表达，而且SPC-A-1中TLR5表达明显增高，这与我们在组织中观察到的结果大致一致。Sławińska A等^[6-8]^发现不同品种的鸡相同器官、组织上TLR5表达不一致，其表达的强度与由生物体的生理状态（尤其与炎症）密切相关，我们发现三种人肺癌细胞株TLR5的表达存在差异，因此推测可能与细胞株的细胞来源不同相关。免疫荧光结果还显示，TLR5在SPC-A-1细胞膜和细胞质均有表达，而且主要表达在细胞膜上，这与既往文献报道^[9, 10]^相符。基于TLR5在NSCLC组织和不同NSCLC细胞株中的表达情况，因此我们选择SPC-A-1细胞株作为实验对象，研究TLR5与特异性配体结合后，其信号通路的活化情况。

TLR在癌细胞的表达增加，可上调NF-κB，产生抗凋亡蛋白，从而促进肿瘤细胞增殖；亦可介导肿瘤细胞释放趋化因子和细胞因子，诱导机体的免疫细胞参与肿瘤细胞微环境中的免疫反应。免疫细胞进一步释放炎性细胞因子、生长因子和血管形成因子，促进肿瘤细胞的生长、侵袭、新的血管淋巴管形成、转移。既往研究^[11]^还发现TLR4、TLR9、TLR7、TLR8等Toll样受体表达在人肺癌细胞株上，与肿瘤细胞的存活与化疗耐药性等相关。Cherfils-Vicini等^[11]^发现TLR7、TLR8可以表达在离体培养的肺癌组织和细胞株上，两者被激活后，NF-κB蛋白、抗凋亡蛋白Bcl-2的表达上调，增加肿瘤细胞的存活及其耐药性。进一步分析发现用配体刺激TLR7或TLR8后，在人肺原发肿瘤细胞和人肺肿瘤细胞株原位检测显示基因表达上调，提示肿瘤细胞受到慢性刺激，说明TLR信号可以直接有促进肿瘤的发生发展。我们选取SPC-A-1细胞株，发现随着鞭毛蛋白浓度的增加，TLR5被激活，其通路下游分子NF-κB荧光素酶的活性增强，且呈浓度依赖性；其活性能被TLR5抗体抑制，随着TLR5抗体浓度的增加，NF-κB荧光素酶的活性逐渐降低，与TLR5抗体浓度呈负相关。说明鞭毛蛋白活化了TLR5通路，并引起下游分子的活化，与既往文献相符。

树突状细胞（dendritic cells, DCs）位于抵御外来入侵第一道防线，接收到警报后可启动后天免疫系统。TLRs位于DCs上，是先天免疫模式识别的主要受体之一，它能识别许多病原微生物上的PAMPs，这些保守序列与TLRs结合后，进而活化机体先天免疫系统，导致一些亲炎症细胞因子的产生，因此TLRs被认为控制着由先天免疫向后天免疫的转变。我们前期研究发现TLR5蛋白不仅在NSCLC组织表达增加，还高表达于NSCLC细胞株中。由于肺癌组织或细胞中其内源性的配体目前并不明确，而鞭毛蛋白是TLR5已知特异的外源性配体，因此我们用鞭毛蛋白刺激TLR5，进一步研究其在NSCLC细胞中激活后，信号通路活化的情况。

TLR与特异PAMPs结合后，自身的异构形态发生改变，衔接分子被募集，下游分子陆续发生磷酸化和/或泛素化，大量蛋白质被募集，或结合，信号通路激活，引起炎性转录因子、炎性基因表达，机体发生免疫防御反应，产生炎症。在此通路中，NF-κB位于TLR下游信号通路的枢纽位置。NF-κB具有多向性调节作用的细胞核转录因子，也是能触发炎症、并广泛存在于炎症反应中的一种关键性因子。NF-κB参与诱导基因表达、调控免疫细胞的激活、肿瘤形成、细胞凋亡、细胞信号转导、炎症反应及多种自身免疫性疾病发生等过程^[12, 13]^，也是炎症和肿瘤的重要枢纽分子。我们检测到用鞭毛蛋白刺激SPC-A-1细胞后，p-IKBα、p-ERK1/2水平均在10 min开始升高，30 min达到高峰，60 min水平明显降低，而相应的的时间点IKBα、ERK1/2蛋白水平并无明显变化，说明SPC-A-1细胞受到鞭毛蛋白刺激后，引起胞浆内关键分子IKBα发生磷酸化，继而降解，引起NF-κB活化移位入胞核，启动下游ERK1/2蛋白磷酸化，及一系列的级联反应。而用鞭毛蛋白刺激TLR5 shRNA细胞后，在0 min、10 min、30 min、60 min四个不同的作用时间点，IKBα、ERK1/2蛋白的水平无明显变化，p-ERK1/2蛋白水平随着时间延长明显增高，而p-IKBα、p-JNK蛋白均未检出。说明TLR5基因并非完全被沉默，表达出的极少量TLR5蛋白可以引起ERK1/2的磷酸化。

NF-κB在肺癌组织中的的表达明显高于正常肺组织，提示在NSCLC的发生发展中可能起重要作用。而且烟雾中的化学物质可引起支气管损伤、支气管炎症，炎症中的一些细胞因子、趋化因子活化NF-κB，进而诱导与癌症相关的基因表达。我们的研究也发现吸烟的肺癌患者中TLR5表达高于非吸烟者，说明吸烟确实提高了支气管炎症的发生。

因此，从我们的实验结果可以推测出鞭毛蛋白刺激NSCLC细胞TLR5活化，通路下游具有活性的磷酸化p-IKBα、p-ERK1/2及p-JNK蛋白表达增多，在30 min达到高峰，NF-κB入核率增加，导致NSCLC细胞活化，但对其生物行为的影响还有待进一步研究。
